# Sleep Disturbance and Metabolic Dysfunction: The Roles of Adipokines

**DOI:** 10.3390/ijms23031706

**Published:** 2022-02-01

**Authors:** Zhikui Wei, You Chen, Raghu P. Upender

**Affiliations:** 1 Department of Neurology, Vanderbilt University Medical Center, Nashville, TN 37232, USA; raghu.p.upender@vumc.org; 2 Department of Biomedical Informatics, Vanderbilt University Medical Center, Nashville, TN 37232, USA; You.chen@vumc.org

**Keywords:** adipokine, cardiometabolic disease, metabolic syndrome, sleep disorder, obstructive sleep apnea

## Abstract

Adipokines are a growing group of peptide or protein hormones that play important roles in whole body metabolism and metabolic diseases. Sleep is an integral component of energy metabolism, and sleep disturbance has been implicated in a wide range of metabolic disorders. Accumulating evidence suggests that adipokines may play a role in mediating the close association between sleep disorders and systemic metabolic derangements. In this review, we briefly summarize a group of selected adipokines and their identified function in metabolism. Moreover, we provide a balanced overview of these adipokines and their roles in sleep physiology and sleep disorders from recent human and animal studies. These studies collectively demonstrate that the functions of adipokine in sleep physiology and disorders could be largely twofold: (1) adipokines have multifaceted roles in sleep physiology and sleep disorders, and (2) sleep disturbance can in turn affect adipokine functions that likely contribute to systemic metabolic derangements.

## 1. Introduction

Adipokines (also known as adipocytokines) are secreted peptides and proteins produced by adipose tissue. They comprise an ever-expanding group of proteins, exemplified by leptin and adiponectin, that play diverse roles in whole body energy metabolism [1]. Adipokines are from different protein families and are produced by diverse cell types, including fat cells, immune cells, and other cells within and outside of the adipose tissue [1]. Studies on adipokines have revealed the importance of adipose tissue as a metabolically active tissue/organ in mediating crosstalk among different tissues and organs [2]. Meanwhile, they have also deepened our understanding of the complex interplay between different physiologic or pathological processes such as metabolism, inflammation, and cardiovascular diseases [2].

Sleep disturbance has been implicated in a wide range of metabolic disorders contributing to significant morbidity and mortality [3]. This has been studied extensively in epidemiological studies in the context of the modern epidemic of metabolic syndrome, a cluster of medical conditions that include central obesity, systemic hypertension, insulin resistance, and dyslipidemia. For example, prospective longitudinal studies in children have shown a temporal relationship between short sleep time and the development of obesity, suggesting a possible causal relationship [4]. In adults, sleep disorders are associated with glucose intolerance, insulin resistance, and a predisposition to type 2 diabetes [5]. Short sleep duration also increases the risk of obesity, hypertension, coronary artery disease, and stroke, and all of which cause morbidity and mortality [6,7].

While the associations between sleep disturbance and metabolic disorders have been repeatedly shown, the underlying mechanisms remain poorly understood. Accumulating evidence suggests that adipokines may provide the link between sleep disturbances and systemic metabolic derangements. This review aims to provide a brief discussion of representative adipokines and their major metabolic functions. Further, we aim to provide a balanced discussion of emerging evidence from both human and animal studies on the role of adipokines in sleep physiology and sleep disorders. These studies collectively demonstrate that the functions of adipokines in sleep physiology and disorders could be largely twofold: (1) adipokines have multifaceted roles in sleep physiology and sleep disorders, and (2) sleep disturbance can in turn affect adipokine functions that are important in systemic metabolic derangements.

## 2. Adipokines in Sleep Physiology and Disorders

Currently, more than 600 adipokines with various biological activities have been described in humans [1]. A common classification scheme roughly divides adipokines into proinflammatory or anti-inflammatory groups [8,9]. It is shown that obesity is associated with the upregulation of proinflammatory adipokines and the downregulation of anti-inflammatory adipokines, leading to the development of chronic low-grade inflammation [8]. This model of pathogenesis was thought to be important in several disease processes, including type 2 diabetes, cardiovascular diseases, dermatological diseases, and osteoarthritis [8,9,10,11]. Comparatively, the roles of adipokines in sleep physiology and sleep disorders are less understood. Recent literature indicates that some adipokines, such as leptin, have direct roles in sleep physiology and sleep disorders. Other adipokines are mainly described as mediators of the metabolic dysfunction of sleep disturbance, including insufficient sleep and obstructive sleep apnea. A selected group of adipokines with demonstrated roles in human sleep physiology and sleep disorders are reviewed below.

### 2.1. Leptin

**Figure 1:**
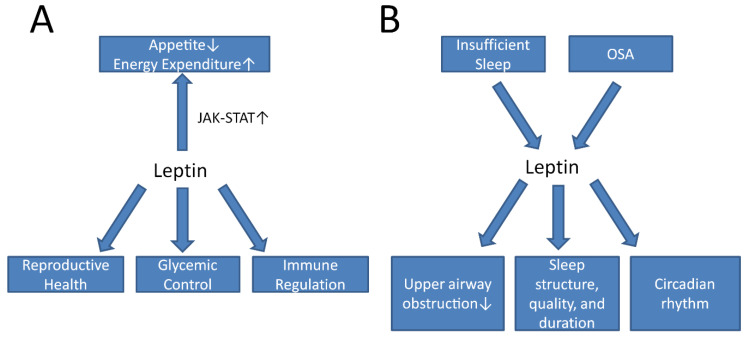
An illustration showing the metabolic and sleep functions of leptin. (**A**) Leptin activates JAK-STAT pathways to decrease appetite and increase energy expenditure centrally. It also has important functions in reproductive health, glycemic control, and immune regulation. (**B**) Insufficient sleep and OSA can both influence leptin levels in the serum. Leptin, in turn, can reduce upper airway obstruction in sleep and improve sleep parameters such as sleep structure, quality, and duration. Leptin may also play a role in circadian rhythms. Abbreviations: JAK, Janus kinase; OSA, obstructive sleep apnea; STAT, signal transducer and activator of transcription.

Leptin is by far the most intensely studied adipokine concerning sleep physiology and sleep disorders (Table 1). Leptin is a peptide hormone produced mainly in the white adipose tissue. Its major function is to regulate whole body energy metabolism by balancing the central pathways that regulate satiety and feeding. It binds to surface Ob receptors (OBRs) in the hypothalamus and activates signaling pathways to regulate satiety and feeding. More specifically, it can activate the JAK-STAT signaling to increase the expression of anorexic peptides such as POMC and CART and inhibit the expression of orexigenic neuropeptides NPY and AgRP to decrease appetite [12]. In addition, it also plays important functions in reproductive health, glycemic control, and bone metabolism (Figure 1A, Table 2) [13].

A number of large population studies have established the importance of leptin in sleep and metabolic functions. The Beijing Child and Adolescent Metabolic Syndrome Study (BCAMS) showed that poor sleep is associated with higher body mass index (BMI), fasting glucose, insulin, and Homeostatic Model Assessment for Insulin Resistance (HOMA-IR) among 3166 school children aged 6–12 [14]. These associations disappeared after adjusting for leptin, underscoring leptin as a potential link between poor sleep and metabolic derangements [14]. Using two cohort studies, involving children in Project Viva (*n* = 655) and adolescents in the Cleveland Children’s Sleep & Health Study (*n* = 502), Boeke et al. showed that chronic insufficient sleep was associated with reduced levels of leptin in girls at age 7 and the association was stronger among girls with greater adiposity [15]. Shorter sleep was also associated with lower leptin levels in adolescent males [15]. These results demonstrated a positive relationship between leptin and sleep time, which were inversely correlated with obesity in the pediatric population.

In adults, the relationship between leptin, sleep time, and BMI is more variable. The Wisconsin Sleep Cohort Study included 1024 participants aged 30–60 and showed a U-shaped curvilinear relationship between sleep duration and BMI, where short sleep duration (<8 h) was associated with reduced leptin, elevated ghrelin, and increased BMI [16]. In a sample of postmenopausal women (*n* = 769, median age 63 years), individuals reporting shorter sleep duration (<6 h) were associated with lower leptin levels, higher energy intake, and lower diet quality [17]. The Quebec Family Study also demonstrated a cross-sectional association between short sleep duration, reduced leptin, and increased adiposity in a sample of 323 men and 417 women aged 21–64 [18]. In contrast, the Cleveland Family Study (CFS) (*n* = 561) showed an increased fasting level of leptin with a reduction in total sleep time and a decrease in rapid eye movement (REM) sleep [19]. The discrepancies of leptin changes associated with short sleep time could be in part due to different characteristics of patient populations (e.g., age, BMI, and prevalence of diabetes and obesity) and different methods of sleep measurement (e.g., polysomnography vs. self-report sleep time). These studies suggest that leptin may have a nonlinear relationship associated with short sleep time modified by other factors such as age, gender, and comorbidities in adults.

Studies involving experimental conditions of sleep, i.e., sleep restriction or sleep loss, provide additional insights into the function of leptin in sleep. Earlier studies involving healthy men showed total sleep loss or sleep restriction attenuated the amplitudes of the diurnal rhythm of leptin [20]. Sleep restriction was also shown to be associated with decreased leptin, increased ghrelin, and increased hunger and appetite in healthy young men [21]. In contrast, subsequent studies showed that acute sleep deprivation or sleep restriction significantly increased morning leptin in healthy women and women with obesity [22,23], as well as in a group of healthy young men and women [24]. These findings suggest there is possibly a gender-dimorphic difference in leptin changes associated with sleep deprivation. Another source of complexity comes from the activation of the stress response, which may be the cause of appetite increase and hormonal changes, not the sleep loss per se [24]. Additionally, caloric intake could also play a role in affecting the leptin changes associated with sleep loss [25].

There is a particular interest in looking at the roles of leptin in sleep disorders among different patient populations with metabolic dysfunction. In a cross-sectional study by Hirota et al. [26], leptin was shown to be positively associated with sleep quality among patients with obesity and type 2 diabetes, but not in nonobese diabetic patients. Interestingly, among patients with anorexia (mean BMI = 13.3) higher levels of leptin and IGF-1 were also associated with longer and deeper sleep [27]. In a group of overweight/obese postmenopausal women, changes in self-reported sleep measures during a 1-year moderate intensity physical activity intervention were not associated with weight loss, nor did it affect ghrelin and leptin levels [28]. Serum leptin levels were associated with BMI in both patients with obstructive sleep apnea syndrome (OSAS) and normal controls. However, only in patients with OSAS, leptin also correlated with the apnea hypopnea index (AHI) [29]. Male patients with OSAS treated with continuous positive airway pressure (CPAP) also showed a significant decrease in leptin levels after 8 weeks of treatment without changes in BMI [30]. These studies indicated that leptin may play a role in ameliorating poor sleep quality and sleep disordered breathing among patients with metabolic derangements, and treatment of sleep disorders such as OSAS can in turn impact leptin as well.

Considerable research has been performed on the relationship between circadian dyssynchrony and metabolic disturbance. Some evidence, particularly those from studies on shift workers, indicates that leptin may also play an important role in mediating the metabolic changes in patients with circadian misalignment/dyssynchrony [31,32,33]. It is noteworthy that leptin expression and serum concentration show circadian rhythmicity, and such circadian changes are more significant in non-obese subjects than obese subjects [31]. Leptin expression is also regulated significantly by behavior cycles, i.e., feeding and sleep, independent of circadian rhythm [32]. Using a 10-day circadian misalignment protocol, Scheer et al. showed that circadian misalignment is associated with decreased leptin, increased glucose and insulin levels, an increase in mean arterial blood pressure, and reduced sleep efficiency [32]. In shift workers such as resident physicians, it was shown that women shift workers with excessive daytime sleepiness have lower levels of leptin than those without sleepiness. Further, the disruption of the normal diurnal sleep pattern and long working hours are also associated with unfavorable lipid profiles and reduced consumption of healthy foods that predispose shift workers to obesity and other metabolic disorders [33]. These studies indicate that leptin may contribute to the development of metabolic dysfunction in shift workers with circadian dyssynchrony.

Studies involving lab animals provide insights into the mechanisms of leptin in sleep physiology. Studies in ob/ob mice (which are genetically deficient in leptin) showed that leptin deficiency reduced ventilatory response to CO_2_ challenges [34] and disrupted sleep architecture and diurnal rhythmicity [35]. Db/Db mice, which are genetically deficient in leptin receptors, demonstrated an increased total sleep time, increased sleep fragmentation, attenuated sleep diurnal rhythmicity, and reduced compensation for acute sleep deprivation [36]. Systemic infusion of leptin in ob/ob mice reversed hypoventilation and reduced the response to CO_2_ challenges [37]. Using ob/ob mice, Yao et al. determined that leptin acts on the forebrain, possibly the dorsomedial hypothalamus, to relieve upper airway obstructions in sleep apnea, and it acts on the hindbrain, possibly the nucleus of the solitary tract, to upregulate ventilatory control [38]. In humans and diet-induced obese (DIO) mice, leptin administered systemically has not been shown to have similar effects in those in ob/ob and Db/Db mice, possibly due to the inhibition of the blood brain barrier [39]. Interestingly, leptin given intranasally significantly improved hypopnea during REM sleep and improved ventilation during REM and non-REM sleep in DIO mice. These effects were independent of the metabolic actions of leptin [39]. Further, leptin concentration was significantly associated with increased compensatory responses to upper airway obstruction in sleep in obese women, and the associations were independent of BMI, waist to hip ratio, neck circumference, and sagittal girth [40]. These findings suggest the leptin likely augments the neurocircuitry to improve disordered breathing in obstructive sleep apnea.

In summary, leptin has been shown to have significant roles in sleep physiology and sleep disorders (Figure 1B). Leptin may represent a component of an important neurocircuitry that can improve hypopnea and reduce upper airway obstruction during sleep. It may have additional functions in regulating sleep structure, duration, and quality, as well as circadian rhythmicity. Additionally, leptin has been shown to link insufficient sleep to increased BMI, fasting glucose, and other metabolic dysfunctions in the pediatric population, though this relationship is less consistent in adult studies. Lastly, leptin can contribute to the metabolic dysfunction in circadian dyssynchrony as well.

### 2.2. Adiponectin

Adiponectin is an adipocyte-specific adipokine with the highest expression of the mature adipocytes. It serves as a critical messenger to mediate the crosstalk between adipose tissue and other metabolic tissues and organs [41]. Functionally, adiponectin binds to surface receptors adipoR1 and adipoR2 and activates downstream signaling pathways such as AMPK and PPARα to increase glucose uptake and fatty acid oxidation (Table 2) [41]. It can also suppress hepatic glucose production via the interaction of the APPL1 and insulin signaling pathway as well as the suppression of gluconeogenic gene expression (Table 2) [41]. In addition, numerous studies have demonstrated its important roles in cardiovascular diseases [41].

Adiponectin plays important roles in sleep physiology and disorders (Table 1). The BCAMS study showed that short sleep duration was associated with decreased levels of the high molecular weight (HMW) adiponectin, but not the full-length adiponectin, among children from 6–12 years old [14]. HMW adiponectin is considered the bioactive component of adiponectin and possesses beneficial effects such as insulin-sensitizing and anti-inflammatory actions [41]. Studies involving smaller cohorts have also shown decreased total adiponectin with reduced sleep time in children [42,43]. Similarly, in a cross-sectional observational study, adiponectin levels were higher in lean Saudi adolescent female subjects (62 lean and 64 obese) aged 14–18 years and increased proportionally to the duration of sleep [43]. These studies indicate that the adiponectin level is positively associated with sleep time in the pediatric population.

In adults, the relationship of adiponectin and sleep varies depending on multiple factors including age, sex, ethnicity, and comorbidities. A study involving six healthy male subjects indicated that adiponectin did not have independent changes in response to the circadian rhythm of sleep [44]. In a group of healthy Japanese male subjects, adiponectin concentration was found to be positively correlated with sleep duration, and the association was found to be stronger in an older age group (>55 years) [45]. Adiponectin levels were also lower in a group of patients with severe OSAS [46]. A recent study also showed that improvement in sleep quality is associated with increased serum adiponectin levels and improved indices of glycemic control [47]. Conversely, chronic sleep deprivation was associated with higher adiponectin levels in a cross-section study among patients with metabolic diseases, after adjusting for age, sex, BMI, hypertension, menopause, and exercise levels [48]. This may reflect a compensatory effect of adiponectin in sleep deprivation among patients with metabolic syndrome [48]. A study involving sleep restriction showed that sleep restriction (five nights of 4 h in bed) led to reduced levels of adiponectin in Caucasian women but an increased level in African American women, with no effect on the adiponectin levels in men, indicating the effect of sleep loss on adiponectin could be sex- and ethnicity-specific [23].

Studies involving lab animals show additional insights into the regulation of adiponectin in sleep. Sleep-deprived 30-day-old male Wistar rats had a reduced expression of adiponectin in retroperitoneal fat without affecting the serum level of adiponectin [49]. Sleep fragmentation during late gestation of mother mice induces epigenetic changes resulting in a long-lasting reduction in adiponectin expression in male offspring mice at 21 weeks when the mother experienced sleep fragmentation for five days [50]. These studies indicate that sleep restriction can have both short-term and long-term effects on the expression of adiponectin.

### 2.3. Chemerin

Chemerin is a newly discovered adipokine shown to have various roles in inflammation, angiogenesis, adipogenesis, and whole body energy homeostasis [51]. Chemerin is highly expressed in the white adipose tissue, liver, and lungs [51]. Chemerin binds to surface receptors CMKLR1 and GPR1 and activates downstream signaling pathways including the MAPK, ERK1/2, and PI3K-Akt pathways to regulate adipogenesis, angiogenesis, and inflammation (Table 2) [51]. In addition, chemerin also regulates glucose metabolism via actions on insulin secretion and the suppression of hepatic glucose production [51]. 

Previous research shows that chemerin may also play an important role in metabolic and sleep disorders. The nocturnal area under the curve (AUC) levels of chemerin were higher among obese/overweight adolescent females than normal weight controls but not in adolescent males, suggesting a gender-dimorphic effect of chemerin in adolescents [52]. Feng et al. showed that serum chemerin levels were significantly elevated in patients with OSAS compared to controls [53]. Serum chemerin levels were also shown to be an independent determinant of OSAS and correlated with the severity of OSAS [53]. Studies by Xu et al. showed that chemerin was higher before and after sleep among patients with severe OSAS compared with the control group after adjusting for BMI [54]. Additionally, chemerin level after sleep was associated with mean SaO_2_%, although plasma chemerin levels were mainly determined by BMI rather than hypoxia during sleep [54]. These studies indicate that chemerin is elevated in proportion to OSAS severity and may be useful as a biomarker for OSAS, as its level correlated with the severity of OSAS (Table 1).

### 2.4. Vaspin

Vaspin is a visceral adipose tissue-derived serine protein inhibitor. In humans, elevated vaspin levels are associated with higher BMI and insulin resistance [55]. Vaspin improves insulin sensitivity in adipose tissue by enhancing Akt signaling [55]. It also reduces endothelial cell inflammation via multiple actions, including reducing the activation of NF-κB [55]. Centrally, vaspin reduces the expression of NPY and increases the expression of POMC to decrease food intake (Table 2) [55]. 

Regarding its role in sleep disorders, studies by Kiskac showed that vaspin was lower in patients with severe OSAS (AHI > 30, *n* = 34) compared to age-matched healthy volunteers (*n* = 25) [56]. In contrast, Pan et al. showed the level of vaspin was markedly increased in the group with obstructive sleep apnea-hypopnea syndrome (OSAHS) compared with healthy controls and positively associated with clinical measures of insulin resistance, including fasting blood glucose, insulin levels, and HOMA-IR [57]. Similarly, in studies by Xu et al., patients with severe OSAS had higher levels of vaspin compared to the control group after adjusting for the BMI, and the levels of vaspin were not associated with any measured sleep parameters [54]. Interestingly, studies by Zhuang showed that healthy male Wistar rats exposed to chronic intermittent hypoxia had higher vaspin mRNA and protein levels in the visceral adipose tissue [58]. It is possible that vaspin is elevated in OSAS secondary to insulin resistance and chronic intermittent hypoxia; however, the exact relationship between vaspin and OSAS warrants further investigation.

### 2.5. Omentin

Omentin is an adipokine expressed in the visceral adipose tissue, including omentum, epicardial fat, and a wide range of other tissues and cells [59]. Omentin has been shown to decrease TNF-α and CRP-induced NF-κb activation and modulate eNOS activity in endothelial cells [60]. Omentin also has been shown to have an insulin sensitizing effect and to promote insulin-stimulated glucose transport via Akt activation in adipocytes in vitro (Table 2) [59].

Regarding its role in sleep (Table 1), studies by Zirlik showed that omentin was increased among 10 patients with newly diagnosed OSAS compared to controls, and CPAP treatment for 3 months reduced the omentin levels toward the levels of the control group [61]. A subsequent study involving 46 newly diagnosed patients with OSAS and 35 controls showed that omentin levels were much higher among patients with OSAS, and its levels correlated with age in patients with OSAS [62]. In contrast, Wang et al. showed that omentin levels were significantly lower among patients with OSAS (*n* = 192) than the healthy controls (*n* = 144). The concentration of omentin was also inversely correlated with AHI among patients with OSAS [63]. A study by Uygur et al. showed omentin levels were significantly lower among patients with OSAS (*n* = 96) when compared with non-apneic controls (*n* = 31), and the levels of omentin were lower among patients with severe OSAS than those with mild/moderate forms of OSAS [64]. Further, they showed that effective treatment with CPAP for 3 months reversed the levels of omentin to the levels of controls [64]. A subsequent study by Zhang et al. showed that the omentin levels were reduced among 30 patients with obesity and severe OSAS compared with healthy controls (*n* = 20) and were associated with sleep parameters such as AHI and SpO_2_ [65]. These studies indicate that omentin is differentially regulated among patients with OSAS, and the direction of change varies among different studies, which could be due to various factors including sample size, patient population, and methodology [66].

### 2.6. Visfatin

Visfatin is an adipokine that is enriched in the human visceral adipose tissue [67]. It is mainly secreted by the macrophages in the adipose tissue rather than by adipocytes [67]. Visfatin is an essential enzyme in nicotinamide adenine dinucleotide (NAD) production and regulates insulin secretion (via actions on syntaxin 4, HNF4a, HNF1b), insulin signaling (via activations on insulin receptor and ERK1/2), and beta-cell function [67]. Visfatin is also shown to be a proinflammatory cytokine and is increased in unstable coronary plaques and involved in endothelial dysfunction, implicating important roles in cardiovascular diseases [67].

In sleep disorders (Table 1), visfatin was shown to be elevated in patients with narcolepsy (*n* = 56) when compared with age- and BMI-matched controls (*n* = 39), mainly driven by HLA DR2-positive narcolepsy patients [68]. Among patients with OSAS, levels of visfatin were positively correlated with sleep latency and negatively correlated with total sleep time, percentage of stage 2 sleep, and REM sleep [69]. Indeed, results from the CFS study showed that there was a 14% increase in visfatin for each hour of reduction in total sleep time and a 31% increase in visfatin for every hour of reduction in REM sleep [19]. Studies by Benedict showed that visfatin levels also displayed a diurnal rhythm in humans, and sleep loss also induced a rhythm phase shift of visfatin that was positively correlated with postprandial glucose levels due to the sleep loss [70]. These studies suggest that visfatin plays a role in regulating sleep structure, and sleep can also regulate whole body inflammation and insulin resistance through adipokines such as visfatin (Table 2).

## 3. Conclusions

**Figure 2:**
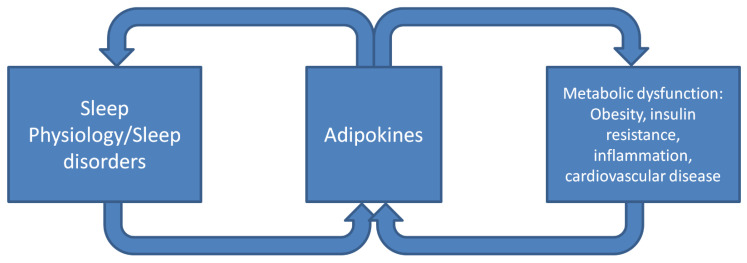
A proposed model of adipokine function in sleep physiology/disorders and metabolic dysfunction. Adipokines have multi-faceted roles in regulating the various aspects of sleep physiology and disorders, which can in turn affect adipokines and their functions, which contribute to systemic metabolic dysfunction.

In this review, we have briefly summarized recent work on representative adipokines and their roles in sleep physiology and disorders. These studies collectively have shown that the functions of adipokines in sleep physiology and disorders could be largely twofold (Figure 2). First, adipokines have multi-faceted roles in sleep physiology and sleep disorders (Table 1). Adipokines such as leptin and visfatin can regulate various aspects of sleep, including latency, duration, quality, and/or structure. Leptin has also been shown to alleviate upper airway obstruction and improve hypoventilation in obese states. This is unique among the adipokines studied to date. Second, adipokines can mediate the metabolic dysfunction associated with sleep disturbance and sleep disorders. Their levels can be differentially regulated in the setting of sleep deprivation, circadian dyssynchrony, and obstructive sleep apnea, contributing to systemic metabolic derangements. Adipokines have been shown to have important roles in the metabolic dysfunction associated with obesity [8]. Given the large overlap between metabolic diseases and sleep disturbance/disorders, adipokines are likely an important mechanism that link poor sleep to systemic metabolic dysfunction. Future large scale prospective studies and additional mechanistic studies of adipokines in sleep physiology and sleep disorders are needed to further clarify the complex interplay between sleep and metabolic function mediated by adipokines.

## Figures and Tables

**Table 1:** Adipokines and their functions in sleep and sleep disorders.

Adipokine	Functions in Sleep and Sleep Disorders
Leptin	↓ in short sleep and obesity (Children) Nonlinear relationship with sleep time (Adults) ↑ in OSAS and correlated with AHI (Adults) Improved sleep quality in adults with obesity and DM2 or anorexia (Adults) Associated with compensatory response in upper airway obstruction in obese women (Adults) Serum levels have diurnal rhythmicity (Adults)
Adiponectin	↓ in short sleep and obesity (Children) Variable relationship with sleep time modified by other factors (Adults)
Chemerin	↑ in OSAS and obesity (adults) Correlated with AHI and mean SaO_2_ in adults with OSAS (Adults) Useful as a biomarker for OSAS (Adults)
Vaspin	Variable changes in OSAS (Adults) Serum levels have diurnal rhythmicity (Adults)
Omentin	Variable changes in OSAS (Adults)
Visfatin	↑ in narcolepsy (Adults) Positively correlated with sleep latency in OSAS (Adults) Negatively correlated with total sleep time, stage 2 sleep, and REM sleep in OSAS (Adults) Serum levels have diurnal rhythmicity (Adults)

List of symbols and abbreviations: ↓: Decreased circulating levels; ↑: Increased circulating levels; AHI: apnea hypopnea index; DM2: diabetes mellitus type 2; OSAS: obstructive sleep apnea syndrome; REM: rapid eye movement (a sleep stage).

**Table 2:** Adipokines and their major molecular pathways and metabolic functions.

Adipokine	Receptor	Major Signaling Pathway	Major Metabolic Functions
Leptin	Ob receptors (OBRs)	JAK-STAT(↑), POMC and CART (↑), NPY and AgRP (↓)	Promote satiety, decrease feeding, improve reproductive health
Adiponectin	AdipoR1, AdipoR2, T-cadherin	AMPK (↑), PPARα(↑), interaction with PI3K/Akt signaling via APPL1, gluconeogenic genes (↓)	Decrease hepatic glucose production, increase fatty acid oxidation, improve insulin sensitivity, improve cardiovascular health
Chemerin	CMKLR1, GPR1, CCrl2	MAPK(↑), ERK1/2 (↑), PI3K-Akt (↑)	Regulate adipogenesis, angiogenesis, and inflammation, decrease glucose production
Vaspin	Unknown	Akt (↑), NF-κB(↓), POMC (↑), NPY(↓)	Improve insulin sensitivity, reduce endothelial inflammation, reduce food intake
Omentin	Unknown	Akt (↑), NF-κB (↓)	Reduce endothelial inflammation, improve insulin sensitivity
Visfatin	Unknown	Syntaxin 4(↑), HNF4α(↑), HNF1β(↑),ERK1/2 (↑)	Regulate NAD production, insulin secretion, and beta-cell function; Increase endothelial dysfunction

List of symbols and abbreviations: ↓: Decreased activity; ↑: Increased activity; AdiopoR1/2, adiponectin receptors 1/2; AgRP, agouti-related peptide; AMPK, AMP-activated protein kinase; APPL1, adaptor protein phosphotyrosine interacting with PH domain and leucine zipper 1; CART, cocaine and amphetamine regulated transcript; CMKLR1, chemerin chemokine-like receptor 1; ERK1/2, extracellular signal-regulated kinase1/2; GPR1, G protein-coupled receptor 1; HNF1β, hepatocyte nuclear factor-1 β; HNF4α, hepatocyte nuclear factor-4α; JAK, Janus kinase; MAPK, mitogen-activated protein kinase; NAD, nicotinamide adenine dinucleotide; NF-kB, nuclear factor k B; NPY, neuropeptide Y; OBR, ob (leptin) receptor; PI3K, phosphoinositide 3-kinase; POMC, pro-opiomelanocortin; STAT, signal transducer and activator of transcription.

## Data Availability

Not applicable.

## Abbreviations

AdiopoR1/2	adiponectin receptors 1/2
AgRP	agouti-related peptide
AHI	apnea hypopnea index
AMPK	AMP-activated protein kinase
APPL1	adaptor protein phosphotyrosine interacting with PH domain and leucine zipper 1
AUC	area under the curve
BCAMS	the Beijing child and adolescent metabolic syndrome study
BMI	body mass index
CART	cocaine and amphetamine regulated transcript
CCrL2	C-C motif chemokine receptor like 2
CFS	the Cleveland family study
CMKLR1	chemerin chemokine-like receptor 1
CPAP	continuous positive airway pressure
CRH	corticotropin- releasing hormone
CRP	C-reactive portein
DIO	diet-induced obesity
DM2	diabetes mellitus type 2
eNOS	endothelial nitric oxide synthase
ERK1/2	extracellular signal-regulated kinase
GPR1	G protein coupled receptor 1
HLA-DR	human leukocyte antigen–DR isotype
HMW	high molecular weight
HNF1β	hepatocyte nuclear factor-1 β
HNF4α	hepatocyte nuclear factor-4α
HOMA-IR	homeostatic model assessment for insulin resistance
HTN	hypertension
IGF1	insulin-like growth factor 1
JAK	Janus kinase
LH	luteinizing hormone
MAPK	mitogen-activated protein kinase
NAD	nicotinamide adenine dinucleotide
NF-κB	nuclear factor κ B
NPY	neuropeptide Y
OBR	obstructive sleep apnea syndrome
OSAHS	obstructive sleep apnea hypopnea syndrome
OSAS	obstructive sleep apnea syndrome
PI3K	phosphoinositide 3-kinase
POMC	pro-opiomelanocortin
PPARα	peroxisome proliferator-activated receptor-α
REM	rapid eye movement
STAT	signal transducer and activator of transcription
TNF-α	tumor necrosis factor-α
